# Diversity and Antimicrobial Activity of Culturable Endophytic Fungi Isolated from Moso Bamboo Seeds

**DOI:** 10.1371/journal.pone.0095838

**Published:** 2014-04-23

**Authors:** Xiao-Ye Shen, Yan-Lin Cheng, Chun-Ju Cai, Li Fan, Jian Gao, Cheng-Lin Hou

**Affiliations:** 1 College of Life Science, Capital Normal University, Beijing, People's Republic of China; 2 Key Laboratory of Bamboo and Rattan Science and Technology of the SFA, International Centre for Bamboo and Rattan, Beijing, People's Republic of China; University of Iowa Carver College of Medicine, United States of America

## Abstract

Bamboos, regarded as therapeutic agents in ethnomedicine, have been used to inhibit inflammation and enhance natural immunity for a long time in Asia, and there are many bamboo associated fungi with medical and edible value. In the present study, a total of 350 fungal strains were isolated from the uncommon moso bamboo (*Phyllostachys edulis*) seeds for the first time. The molecular diversity of these endophytic fungi was investigated and bioactive compound producers were screened for the first time. All the fungal endophytes were categorized into 69 morphotypes according to culturable characteristics and their internal transcriber spacer (ITS) regions were analyzed by BLAST search with the NCBI database. The fungal isolates showed high diversity and were divided in Ascomycota (98.0%) and Basidiomycota (2.0%), including at least 19 genera in nine orders. Four particular genera were considered to be newly recorded bambusicolous fungi, including *Leptosphaerulina*, *Simplicillium*, *Sebacina* and an unknown genus in Basidiomycetes. Furthermore, inhibitory effects against clinical pathogens and phytopathogens were screened preliminarily and strains B09 (*Cladosporium* sp.), B34 (*Curvularia* sp.), B35 (undefined genus 1), B38 (*Penicillium* sp.) and zzz816 (*Shiraia* sp.) displayed broad-spectrum activity against clinical bacteria and yeasts by the agar diffusion method. The crude extracts of isolates B09, B34, B35, B38 and zzz816 under submerged fermentation, also demonstrated various levels of bioactivities against bambusicolous pathogenic fungi. This study is the first report on the antimicrobial activity of endophytic fungi associated with moso bamboo seeds, and the results show that they could be exploited as a potential source of bioactive compounds and plant defense activators. In addition, it is the first time that strains of *Shiraia* sp. have been isolated and cultured from moso bamboo seeds, and one of them (zzz816) could produce hypocrellin A at high yield, which is significantly different from the other strains published.

## Introduction

Bamboos are well-known for their therapeutical effects and potential health benefits. They are used as bioactive agents for a variety of applications, including bamboo charcoal (bintochan), bamboo vinegar, bamboo juice, bamboo beer, bamboo salt, and tender shoot used in Chinese cuisine. There are also many traditional drugs associated with bamboos for treating fever and detoxification which have been used in Indian Ayurvedic medicine and Chinese herbal medicine since ancient times.

Moso bamboo (*Phyllostachys edulis* (Carr.) H. De Lehaie), a member of Bambusoideae (Poaceae), is one typical vegetative, monopodial bamboo species, and native to the subtropics of China. Because of giant size, high production, various uses and wide distribution, it has long been considered as the most important economic bamboo species in China. However, there is a considerable ecological problem in moso bamboo, as their flowers appear only once every 60–120 years, followed by the death of the flowered culms [1]. Sexual propagation plays a vital part in the sustainable production of moso bamboo, but the seeds are uncommon and have a low germination rate [2], [3]. In particular, seed germination of moso bamboo is often associated with high fungal contamination [4] and some fungal endophytes have serious negative effects on the seed survival in tissue culture [5]. It has been demonstrated that bamboo seeds colonized by field and storage fungi, could be a source of potential pathogens, which might pose problems in nurseries [6], [7].

Endophytic fungi colonize almost all plants and have been isolated from all plant parts such as roots, stems, leaves, barks, floral organs and even seeds [8]–[11]. The relationships between the endophytic fungi and their hosts may be saprophytic, pathogenic or even mutualistic [12], [13]. Diverse endophytes have been investigated in seeds of several hosts, of which most (>90%) belong to Dothideomycetes and Sordariomycetes [8], [14]. The seed-associated fungal endophytes were usually implicated in assisting seeds in germination of seed pods but only for a short time due to weather conditions and posing problems in nurseries [5], [15]. Fungal endophytes in the tissues of bamboos have also been identified as species from Dothideomycetes and Sordariomycetes, and their molecular diversity has been analyzed based on internal transcriber spacer (ITS) region sequences of the ribosomal DNA [16], [17]. However, the seed-associated endophytes from moso bamboo have not yet been investigated.

Some bambusicolous fungi also have medicinal effects, similar to their host's or even more effective. *Polyporus mylittae* Cooke. et Mass., *Ganoderma lipsiense* (Batsch) G. F. Atk. and *Dictyophora indusiata* (Vent.) Desv. are all well-known edible macrofungi [18], and have been used as ‘herbal’ treatments for various human diseases in China for over 1000 years [19]–[21]. Cytochalasin C and neoengleromycin from *Engleromyces sinensis* M. A. Whalley. et al., and hypocrellins from *Hypocrella bambusae* (Berk. & Broome) Sacc. and *Shiraia bambusicola* P. Henn., are active ingredients from medicinal macrofungi associated with bamboos, which display broad-spectrum activity against clinical microorganisms, viruses and tumor cells [22]–[26].

The aims of the present study were firstly to produce a sequence-based estimate of the diversity of culturable endophytes in moso bamboo seeds and their isolation frequencies. In addition, the bioactivities of these fungi against pathogenic microorganisms were investigated and the effective metabolites from these endophytes were examined.

## Materials and Methods

In our study, the materials are only referred to the collection of moso bamboo seeds and no specific permissions are needed for the process. There are three noticeable reasons for this case:

1. Moso bamboo would die after flowering, so the traditional method was to collect seeds from the flowered plants;
2. Moso bamboo is a typical vegetative bamboo species, and because of high production, various purposes and wide distribution, it has long been considered as the most important economic bamboo species in China. Moso bamboo is not endangered or protected species in our country.
3. There are large-area artificial forests of stock plant transplantation for moso bamboo in China, so the seed collection didn't need specific permissions.

### Collection of Seeds and Isolation of Fungal Endophytes

Fresh and healthy seeds were collected from moso bamboo in one plantation (110° 17′∼110° 47′ E,25° 04′∼25° 48′ N) in Guilin City in the Guangxi Zhuang Autonomous Region in China. More than 100 seeds were randomly selected for fungal isolations. They were surface-sterilized by 75% ethanol for 30 seconds, 5% NaOCl for 10 min, and rinsed by sterile water [16]. After sterilization, each seed was cut into three fragments and these samples were individually planted onto 2% potato dextrose agar media (PDA, containing (g/L): potato 200, dextrose 20 and agar 20; pH 6.0.), at 20°C without light. The fungal cultures isolated from seeds were recorded and deposited in the China Forestry Culture Collection Center (CFCC). Colony characteristics, including color (surface and reverse), elevation, texture, type of mycelia, margin shape, and density of mycelia on the medium were examined after 1∼3 weeks of incubation.

### DNA Extraction, Amplification, Sequencing and Molecular Identification

Fungal mycelia from subcultured colonies were scraped from the surface of the agar and frozen at −20°C for one night for the extraction of DNA. Extractions were performed using E.Z.N.A.™ Fungal DNA Mini Kit (Omega Biotech, Norcross, United States) and the target regions of ITS rDNA were amplified by ITS1-F/ITS4 [27]. The PCR mixture (25 µL, total volume) contained 0.5 µL template, 0.5 µL of each primer (25 µM each), 12.5 µL 2× MasterMix (including 10× buffer, dNTPs and Taq polymerase) and ddH2O (Tiangen, Beijing, China). Thirty-five cycles consisting of denaturation at 94°C (30 s), annealing at 50°C (45 s) and extension at 72°C (60 s) were run and the final extension step at 72°C for 7 min was performed using Techne TC-512 (Keison Products, Beijing, China). Finally, the purified amplicons were sequenced by Invitrogen Biotechnology Co. Ltd. (Beijing, China). To identify the isolates, sequences were subjected to a BLAST search with the NCBI database (http://www.ncbi.nlm.nih.gov/). Only matches of sequences published in journals were used. Priority was given to sequences derived from authoritative materials, such as ex-type or ex-epitype cultures. The sequences of the present study were also deposited at GenBank.

### Fungal Culture and Crude Preparation

Endophytic fungi isolates were cultured in PDA media. The fresh mycelia of different endophytic fungi were grown on plates at 25°C for more than 7 d. Five plugs (6 mm in diameter) of growing culture plus the adhering mycelium were subsequently added to 250 ml Erlenmeyer flasks containing 100 ml of Potato Dextrose Broth media (PDB, containing (g/L): potato 200 and dextrose 20; pH 6.0). All liquid cultures were kept at 25°C for 7–10 d with shaking (150 rpm). The fermentation of each fungus was filtered to separate the filtrates from the mycelia. The mycelia and filtrates were separately extracted with ethyl acetate (EtOAc) in order to obtain mycelial and filtrated extracts [28].

### Agar Diffusion Assay

The endophytic fungi were screened using the agar diffusion method, as a rapid and qualitative selection of the bioactive microorganisms. Endophytic fungi were cultured on PDA media at 25°C over 7 d. Agar plugs (6 mm in diameter) of growing culture plus the adhering mycelia were subsequently added to Luria Broth Agar media (LBA, containing (g/L): tryptone 10, yeast extract 5, NaCl 10 and agar 20; pH 6.0) and PDA media, supplemented with 0.5% olive oil previously spread with bacteria (*Staphylococcus aureus*, *Bacillus subtilis*, *Listeria monocytogenes* and *Salmonella bacteria*) and yeasts (*Rhodotorula rubra*, *Saccharomyces cerevisiae* and *Candida albicans*). The cultures of bacteria and yeasts were also deposited at CFCC. Plates were incubated at 37°C for 24 h for the bacteria and 28°C for 2–7 d for the yeasts. The inhibition zones around the agar plugs were measured to record the antimicrobial activity of fungal isolates [29].

### Disk Diffusion Assay

The antifungal activities of fungal extracts were tested in a number of pathogenic fungi: *Curvularia eragrostidis*, *Pleospora herbarum*, *Arthrinium sacchari*, *Arthrinium phaeospermum* and *Phoma herbarum*. These cultures of fungi were all deposited at CFCC. The bioactive extracts of mycelia and filtrates were assessed for antimicrobial activity by the disc diffusion method at a concentration of 100 µg/disk. Antimicrobial activity against pathogenic fungi was estimated by the size (diameter in mm) of growth inhibition zones. Each inhibition assay was repeated three times, and analysis of variance was conducted by SPSS 18.0 for Windows (SPSS Inc., Chicago, USA).

## Results

### Isolates, Sequence Data and Diversity

A total of 350 fungal isolates were designated into 69 morphotypes based on cultural characteristics. Sequences of ITS region were generated for the isolates from each morphotype (69 in total). ITS sequences were compared with those deposited in GenBank using a BLAST search (http://www.ncbi.nlm.nih.gov/), and directly with sets of authentic sequences from published studies of taxa (Table S1). The results show that all 350 isolates represented at least 19 genera (Table 1). The majority of ITS sequences from the fungal isolates did not show complete sequence identity with sequences present in GenBank, ranging from 0.2% to greater than 10% sequence variation. All the ITS sequences have been deposited in GenBank, and the accession numbers are HQ654261 and HQ696018∼85 corresponding to individual isolates (Table S1).

**Table 1:** Number of endophytic fungi isolated from moso bamboo seeds and the frequency of colonization (FC%).

Genus (when stated in GenBank)	Phylum; Subclass; Order;	Strain	Isolates number	FC%
*Cladosporium*	Ascomycota; Dothideomycetes; Capnodiales	B01, B05, B06, B08, B09, B10, B11, B25, zzz409, zzz1737	84	24.00
*Aureobasidium*	Ascomycota; Dothideomycetes; Dothideales	B23	2	0.57
*Alternaria*	Ascomycota; Dothideomycetes; Pleosporales	zzz407, zzz1740	12	3.43
*Curvularia*	Ascomycota; Dothideomycetes; Pleosporales	B34	2	0.57
*Leptosphaerulina*	Ascomycota; Dothideomycetes; Pleosporales	zzz511	6	1.71
*Phoma*	Ascomycota; Dothideomycetes; Pleosporales	B29, zzz202	18	5.14
*Shiraia*	Ascomycota; Dothideomycetes; Pleosporales	B02, B17, B18, B22, B27, B33, zzz510, zzz613, zzz815, zzz816, zzz1021, zzz1023, zzz1225, zzz1226	66	18.90
undefined genus 1	Ascomycetes; Dothideomycetes; Pleosporales	B35, zzz1429, zzz1632	10	2.86
undefined genus 2	Ascomycota; Dothideomycetes;	zzz714	1	0.29
*Penicillium*	Ascomycota; Eurotiomycetes; Eurotiales	B19, B32, B38	6	1.71
*Fusarium*	Ascomycota; Sordariomycetes; Hypocreales	zzz101, zzz305a, zzz612, zzz818, zzz1124, zzz1327, zzz1739	48	13.70
*Simplicillium*	Ascomycota; Sordariomycetes; Hypocreales	B26	2	0.57
*Colletotrichum*	Ascomycota; Sordariomycetes; Phyllachorales	B12, B21, B24, B31, B37, zzz303, zzz305, zzz920, zzz1428, zzz1633, zzz1738, zzz1943	54	15.43
*Arthrinium*	Ascomycota; Sordariomycetes; Xylariales	B16, B28, zzz304, zzz1022, zzz1530, zzz1842	24	6.85
*Monographella*	Ascomycota; Sordariomycetes; Xylariales	B13	4	1.14
*Pestalotiopsis*	Ascomycota; Sordariomycetes; Xylariales	zzz2045	2	0.57
*Xylaria*	Ascomycota; Sordariomycetes; Xylariales	zzz1741	2	0.57
*Sebacina*	Basidiomycota; Agaricomycetes; Sebacinales	zzz919	6	1.71
undefined genus 3	Basidiomycota; Basidiomycetes	zzz1735	1	0.29

For the further taxonomic analysis, the ITS sequences of the 69 representative isolates were aligned with reference sequences from GenBank based on sequence similarity, or because they were potentially related taxa. The sequence alignments indicated that the isolates belonged to the phyla Ascomycota and Basidiomycota, corresponding to nine orders (Table 1). In the Basidiomycota, isolate zzz919 was close to *Sebacina endomycorrhiza* (Sebacinaceae, Sebacinales, Agaricomycetes) and isolate zzz1735 was placed in the Basidiomycetes without any similar defined sequence at the generic level. Within the Ascomycota (Table 1), three classes (Dothideomycetes, Eurotiomycetes and Sordariomycetes) were included and at least seven orders (Capnodiales, Dothideales, Pleosporales, Eurotiales, Hypocreales, Phyllachorales and Xylariales) were detected from the fungal isolates. Ten strains (B01, B05, B06, B08, B09, B10, B11, B25, zzz409 and zzz1737) belonged to the genus *Cladosporium* in the Capnodiales, and only one (B23) to *Aureobasidium* in the Dothideales. Twenty-three isolates belonging to the Pleosporales were placed respectively in the genus *Alternaria* (isolates zzz407 and zzz1740), *Curvularia* (isolate B34), *Leptosphaerulina* (isolates zzz511), *Shiraia* (16 isolates, including zzz1226, zzz1632, zzz1225, B18, B33, B02, zzz816, zzz815, zzz613, B27, B22, zzz510, zzz1023, zzz1021, B17 and zzz1740) and undefined genus 1(three isolates, including B35, zzz1429 and zzz1632).

One isolates (zzz714) was affiliated to the Dothideomycetes, but the published NCBI reference sequence was not identified at order level. Three isolates (B19, B32 and B38) were species of *Penicillium* (Eurotiales, Eurotiomycetes) with well supported sequence alignment. Sordariomycetes contained three orders (Hypocreales, Phyllachorales and Xylariales), seven genera (*Fusarium*, *Simplicillium*, *Colletotrichum*, *Arthrinium*, *Monographella*, *Pestalotiopsis* and *Xylaria*) and 29 culturable strains. Seven of them (zzz101, zzz305a, zzz612, zzz818, zzz1124, zzz1327 and zzz1739) represented taxa from the dominant genus *Fusarium* (Hypocreales). Another isolate (B26) was close to *Simplicillium* (Hypocreales) with high similarity. Phyllachorales contained 12 isolates (B12, B21, B24, B31, B37, zzz303, zzz305, zzz920, zzz1428, zzz1633, zzz1738 and zzz1943) with high similarity, all of which were close to several species of *Colletotrichum*. In Xylariales, isolates B16, B28, zzz304, zzz1022, zzz1530 and zzz1842 were analogous with *Arthrinium* species, and isolates B13, zzz2045 and zzz1741 were assigned to *Monographella*, *Pestalotiopsis* and *Xylaria*, with high identities, respectively.

Three hundred and forty-three isolates belonged to the Ascomycota (98.0% frequency) and only seven to the Basidiomycota (2.0% frequency), representing at least 19 genera in nine orders (Table 1). Pleosporales was the most frequent order (32.61%) and had the most genera (six genera) in this study, while *Cladosporium* in Capnodiales was the most frequent genus (24.0%). The seven orders in the Ascomycota belonged to the Dothideomycetes (57.47% frequency), Eurotiomycetes (1.71% frequency) and Sordariomycetes (38.83% frequency).

### Detecting Antimicrobial Activities of the Culturable Strains

The more important purpose of the present study was to investigate the antimicrobial activity of the culturable fungi associated with bamboo seeds and screen the bioactive strains which might have applied potentials. There were 69 representative endophytes from moso bamboo seeds, which were screened by agar diffusion assay, to confirm if they demonstrated antimicrobial activities against clinical pathogens. The tested micro-organisms included model bacteria (*S. aureus*, *B. subtilis*, *L. monocytogenes* and *Salmonella* sp.) and yeasts (*C. albicans*, *R. rubra* and *S. cerevisiae*). The preliminary evaluation demonstrated that the various fungal isolates displayed different antimicrobial effects (Table 2). Endophytic fungi strain B09 inhibited the growth of two human pathogenic bacteria *S. aureus* and *B. subtilis* and also displayed good activity against *C. albicans*. Strain B34 had effect on four clinical microorganisms (*B. subtilis*, *L. monocytogenes*, *Salmonella* sp. and *C. albicans*) and B35 was also active against two bacterial species (*S. aureus* and *B. subtilis*) and two fungal species (*C. albicans* and *R. rubra*). Strain B38 displayed the widest spectrum of anti-microorganisms (six species – *S. aureus*, *B. subtilis*, *L. monocytogenes*, *Salmonella* sp., *C. albicans* and *R. rubra*) and had the strongest activity against three of them (*S. aureus*, *B. subtilis* and *C. albicans*), as well as strain zzz816. However isolate zzz816 showed higher activity against *R. rubra* in the antifungal assay and less inhibitory effect on *L. monocytogenes* and *Salmonella* sp. in the antibacterial test. There was no fungal endophyte with distinct bioactivity against *S. cerevisiae*.

**Table 2:** Antimicrobial activity of fungal isolates from moso bamboo seeds against human pathogens.

Isolate No.	*Staphylococcus aureus*	*Bacillus subtilis*	*Listeria monocytogenes*	*Salmonella bacteria*	*Candida albicans*	*Rhodotorula rubra*	*Saccharomyces cerevisiae*
B09	+	+	−	−	++	−	−
B34	−	+	+	+	+	−	−
B35	+	+	−	−	+	+	−
B38	+++	+++	++	++	+++	+	−
zzz816	+++	+++	+	−	+++	++	−

−: no activity (<10 mm); +: activity (12–15 mm); ++: good activity (15–20 mm); +++: very good activity (>20 mm).

Of 69 representative isolates, five strains (B09, B34, B35, B38 and zzz816) had inhibitory effects on at least four of the pathogenic micro-organisms tested, and these were selected to continue in the bioactive compounds screening. To test the bioactivity of the endophytic isolates against plant pathogenic fungi, five pathogenic bambusicolous fungi (*Curvularia eragrostidis, Pleospora herbarum, Arthrinium sacchari, Arthrinium phaeospermum* and *Phoma herbarum*) were selected for the further antagonism test [4]. Bioactivity of extracts from endophytic fungi were estimated from the size (diameter in mm) of growth inhibition zones (DGI), which is an indication of the efficacy of antifungal activity, and the effect of ethyl acetate extracts was tested at 100 µg/ml against pathogens. Comparing crude extracts of mycelia and filtrates, the variations in the calculation of DGI of five endophytic fungi displayed the same trend (Table 3), but ethyl acetate extracts of mycelia showed higher growth inhibition than the related filtrates. In the disk diffusion test, none of the crude extracts were found to be effective against *Curvularia eragrostidis* and *Phoma herbarum*, and strain B35 didn't exhibit antifungal activity distinctly against any of the plant pathogens, either from mycelia or filtrates.

**Table 3:** Antifungal activity of ethyl acetate extracts of the mycelia and filtrates of endophytic fungi from moso bamboo seeds tested by disk diffusion assay^a^.

	Inhibition zone (mm±SD)^b^				
Isolate No.	*Curvularia eragrostidis*	*Pleospora herbarum*	*Arthrinium Sacchari*	*Arthrinium Phaeospermum*	*Phoma herbarum*
DMSO	-	-	-	-	-
B09^M^	-	13.72±0.41^g^	10.38±0.59^e^	9.74±0.31^e^	-
B09^F^	-	9.85±0.13^e^	7.99±0.23^cd^	7.93±0.25^cd^	-
B34^M^	-	7.86±0.23^cd^	8.07±0.18^cd^	8.06±0.32^cd^	-
B34^F^	-	-	-	-	-
B35^M^	-	-	-	-	-
B35^F^	-	-	-	-	-
B38^M^	-	11.90±0.07^f^	14.07±0.14^gh^	13.83±0.07^gh^	-
B38^F^	-	7.35±0.51^c^	8.42±0.45^d^	9.80±0.20^e^	-
zzz816^M^	-	11.80±0.29^f^	15.09±0.42^i^	14.09±0.26^gh^	-
zzz816^F^	-	8.22±0.16^cd^	9.53±0.27^e^	10.39±0.13^e^	-

a Diameter of growth inhibition in mm.

“-”  =  not active.

b mm±SD: millimeter ± standard deviation.

M Ethyl acetate extracts of the mycelia.

F Ethyl acetate extracts of the filtrates. Statistical analysis of the data was performed with SSPS 18.0 using Student-Newman-Keuls test for determining significant difference (α = 0.05).

cd low activity, ^ef^moderate activity, ^ghi^high activity.

Ethyl acetate extracts of B09 were found to be the most effective agents, against the widespread plant pathogenic fungus *Pleospora herbarum*, both from mycelia and filtrates. With bioactivity against *Arthrinium sacchari*, DGI of extracts from zzz816 were significantly higher than the others under the same conditions. From the calculation of DGI, the mycelial and filtrated extracts of B38 and zzz816 had the most marked activity against *Arthrinium phaeospermum*.

Strain B38 and zzz816 displayed the same broad-spectrum of bioactivity against bambusicolous pathogens, but extracts of B09 inhibited the growth of *Pleospora herbarum* more significantly, and others more weakly. Extracts of B34 had low activity against plant pathogens, with no measureable effect from B35 either.

## Discussion

The analysis of ITS region revealed that a remarkable diversity of fungal endopytes from moso bamboo seeds was mainly distributed in Dothideomycetes and Sordariomycetes. Many species in the two subclasses have been described from bamboos, including saprophytes, pathogens and endophytes [4], [16], [30], [31]. Previously, a total of 65 fungi belonging to 37 genera have been reported on stored seeds of different species of bamboos [4]. In this study, we obtained at least 10 genera in Dothideomycetes, seven genera in Sordariomycetes, one in Eurotiomycetes and two in Agaricomycetes as endophytes from moso bamboo seeds, and some of them were reported from bamboo seeds or moso bamboo for the first time.

At least 12 genera in the present study have been reported as pathogens and endophytes, and in order of frequency, they were *Cladosporium* (24.0%), *Shiraia* (18.90%), *Colletotrichum* (15.43%), *Fusarium* (13.70%), *Arthrinium* (6.85%), *Phoma* (5.14%), *Alternaria* (3.43%), *Penicillium* (1.71%), *Aureobasidium* (0.57%), *Curvularia* (0.57%) and *Xylaria* (0.57%) (Table 1).

Four of them, *Fusarium*, *Arthrinium*, *Alternaria* and *Aureobasidium*, have been reported as pathogens of moso bamboo [32]–[34]. Other than *Aureobasidium*, they have previously been reported on bamboo seeds [4], [35] and several species, including *F. pallidoroseum*, are seed-borne and capable of causing infection of emerging seedlings [4]. Isolate B23 was highly similar to *Aureobasidium pullulans* by ITS sequence (only 2% difference). This is a common fungus on all plant material and *A. pullulans* has been reported on moso bamboo in China in one previous study [34].

Another three genera (*Cladosporium*, *Phoma* and *Curvularia*) have been reported as pathogens of some bamboo species excluding moso bamboo, and they were also associated with seeds of some bamboo species [4]. These genera were reported from moso bamboo for the first time as far as the authors are aware.

*Penicillium* sp. has been documented to infect seeds of *Bambusa nutans* in India and Thailand [4], [35]. Six isolates obtained during the study by Mohanan [4] and Shukla et al. [35] shared high similarity with three species of *Penicillium* (3% difference in ITS sequences) in the present study (Table S1 and Table 1).

Species of the other three genera, *Shiraia*, *Colletotrichum* and *Xylaria*, are common pathogens of bamboos, but have not yet been reported on moso bamboo seeds [33], [36], [37]. A total 122 isolates (34.86%) from moso bamboo seeds in our study were close to some species of these genera. One exception was isolate zzz1740 as the generic position was undefined in the present study, with a 5% sequence variation. Isolate B17 was obviously close to *Shiraia* sp. by sequence alignment, but was proved to be far from *S. bambusicola* and other *Shiraia*-like fungi with 5% base pair differences based on ITS [38].

Five taxa isolated with low frequency in our study had not yet been reported as pathogens of bamboos. *Pestalotiopsis* (0.57%) have been reported as endophytes [16]. *Leptosphaerulina* (1.71%), *Simplicillium* (0.57%), *Sebacina* (1.71%) and an undetermined genus (2.86%) were new bambusicolous fungi which have previously been reported as pathogens of other plants [39]–[41]. Actually, the similarity of the ITS sequences was a little low, especially as isolate zzz919. This isolate had 18% difference of ITS sequence with *Sebacina endomycorrhiza* (HQ696070). To determine these unknown taxa, such as zzz714 (0.29%) and zzz1735 (0.29%), further studies of other conservative genes and morphology are needed.

In tropical humid areas such as the Guangxi Zhuang Autonomous Region, bamboo seeds have been reported to be particularly vulnerable to several field and storage fungi, and many of these fungi are potential pathogens [6], [7]. Many of the seed-associated endophytes might affect the viability of seeds and pose problems in nurseries [4]. To date, far more than 1100 species of bambusicolous fungi are known, with only a few previously known from bamboo seeds, including endophytic fungi [4], [30], [35], [42]. The sampling of the present study focused on the diversity of endophytes from moso bamboo seeds in China. The results showed that at least 19 genera of endophytes were identified. It was difficult to define all taxa at species level. Some of the taxa were identified according to the accepted generic variation at species level after comparing these to the taxa by ITS sequences in published references. Several taxa could be identified only to the family, order or subclass level [9]. Furthermore, the endophytic diversity in this study presumably only accounts for a fraction of the total diversity within this one plantation of moso bamboo.

The other purpose of the present study was to investigate the antimicrobial activity of the culturable fungi associated with bamboo seeds and screen the potentially useful bioactive strains. Of 69 fungal isolates, B09 (*Cladosporium* sp.), B34 (*Curvularia* sp.), B35 (undefined genus 1), B38 (*Penicillium* sp.) and zzz816 (*Shiraia* sp.) displayed broad-spectrum activities against human pathogenic bacteria (*S. aureus*, *B. subtilis*, *L. monocytogenes* and *Salmonella* sp.) and clinical yeasts (*R. rubra*, *S. cerevisiae* and *C. albicans*) by the agar diffusion method. Furthermore, the crude extracts from five endophytic fungi also exhibited differences in the extent of antimicrobial activity against bambusicolous pathogenic fungi (*A. Sacchari*, *A. Phaeospermum*, *C. eragrostidis*, *Pleospora herbarum* and *Phoma herbarum*). In particular, B09, B38 and zzz816 showed broad-spectrum and effective bioactivity in antagonistic tests, and these will be tested as potential biocontrol agents in further studies.

**Figure 1:**
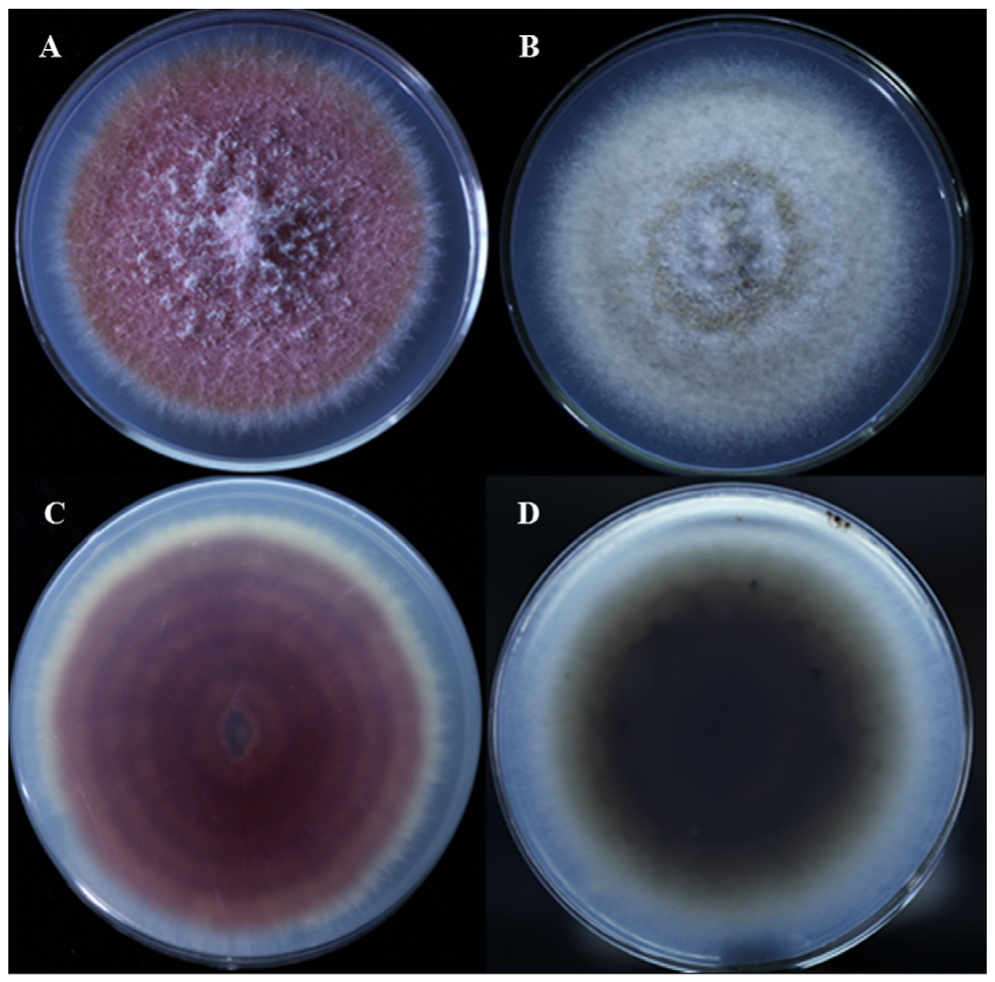
Colony morphology of isolates of *Shiraia* sp. on PDA media. **A**. Upper colour of colony from isolate 816 (Endophytic fungi from moso bamboo seeds); **C**. Reverse colour of colony from isolate 816; **B**. Upper colour of colony from isolate of fruit body of *S. bambusicola*; **D**. Reverse colour of colony from isolate of fruit body.

It is noticeable that isolate zzz816 was closely related to *Shiraia* species, a known bambusicolous fungus in East and Southeast Asia. This fungus is mainly found on *Brachystachyum densiflorum* and related species in China and *Bambusa* species in Japan [36], [43]. There is no information about *S. bambusicola* in the Guangxi Zhuang Autonomous Region. Interestingly, fruiting bodies of this fungus frequently occur on *B. densiflorum* in China, while they don't usually appear on *P. edulis* or other *Phyllostachys* spp. and as endophytes on *Take* and *Sasa* species [16]. It is hypothesized that this fungus can live on various bamboos as asymptomatic endophytes without producing fruiting bodies, due to limiting conditions such as nutrition or host structure. The fruit body of *S. bambusicola* has been used in traditional medicine in China, and its compounds have been found to be useful for antitumor activity and antiangiogenesis [36]. Hypocrellins, as the dominant effective compounds of *S. bambusicola*, have attracted a great deal of attention because of their light-induced antifungal, antiviral and antitumor activities. It is crucial to enhance the production of this compound for future research and therapeutic applications [44], [45]. The paclitaxel (taxol), was well-known for the clinical application against different types of cancer, but the low extraction efficiency (0.0074%) has highly restricted the corresponding development [46]. To break the bottleneck of production, there were many endophytic fungi of *Pestalotiopsis* isolated from yew trees, and some of them have been applied to improving taxol yield significantly [47]. Similarly, in contrast to the traditional resource of the fruiting body, one hypocrellin-producing strain zzz816 (*S. bambusicola*) was isolated from the moso bamboo seeds, and it was significantly different from the original strains in the previous reports by the colony colour (Figure 1). In a preliminary test, zzz816 exhibited the highest content of hypocrellins among all the unmodified strains as far as is known and it is believed that the production efficiency of the active agent would be improved tremendously by breeding of novel industrial mutants and optimization of the fermentation process in the further research.

The species of *Cladosporium* (B09), *Curvularia* (B34) and *Penicillium* (B38) have all been recorded as widespread strains among plants as endophytes, saprophytes or pathogens and there are many bioactive agents with antiviral, antifungal and antitumor activities from these corresponding species [48]–[53]. Potentially all three fungal isolates from moso bamboo seeds could be a source of original chemical products. Strain B35 was closely related to species of Pleosporales isolated from plants as endophytes, but the specific genus could not be identified by the ITS sequence alignment. Further studies of sequencing gene SSU and a rapid analytical method based on reverse-phase high-performance liquid chromatography were in progress to confirm the taxonomic status of the endophytic fungi and identify new and useful bioactive agents from these undiscovered species.

This is the first report analyzing the diversity of fungi from moso bamboo seeds, and the presented results could contribute to the understanding of the ecological role of bambusicolous fungi. Furthermore, screening of all the fungal isolates for biological activity demonstrated that five endophytic strains (B09, B34, B35, B38 and zzz816), had potential agricultural and pharmaceutical applications.

Strain zzz816 was isolated from moso bamboo seeds as fungal endophytes, and produced high-yield hypocrellins. Unlike hypocrellin biosynthesis strains generally originating from the fruiting body of *S. bambusicola*, this study suggests that it might be feasible to enhance the efficiency of industrial hypocrellin production using high-yield strains on the selection of novel plantations, and the future development of fermentation product yields would be improved furtherly at the base of strains breeding and process optimization.

## Supporting Information

Table S1 **Taxon designation of fungal endophytes from moso bamboo seeds based on sequence data from the internal transcribed spacer regions of nuclear ribosomal DNA (ITS rDNA).**
(DOC)
