# Supplementary material for: Diversity and Antimicrobial Activity of Culturable Endophytic Fungi Isolated from Moso Bamboo Seeds
Source: PLoS One. 2014 Apr 23;9(4):e95838. doi: 10.1371/journal.pone.0095838 (PMC3997407; doi:10.1371/journal.pone.0095838)
Supplement: Table S1 — Taxon designation of fungal endophytes from moso bamboo seeds based on sequence data from the internal transcribed spacer regions of nuclear ribosomal DNA (ITS rDNA). (DOC) [file pone.0095838.s001.doc]

**Table S1.** Taxon designation of fungal endophytes from Moso bamboo seeds based on sequence data from the internal transcribed spacer regions of nuclear ribosomal DNA (ITS rDNA).

| **Genus (stated in GenBank)** | **Phylum; Subclass; Order;** | **Strain** | **ITS (No.)^a^** | **Most closely related species** | **% similarity** | **Reference ITS (No.)^b^** |
| --- | --- | --- | --- | --- | --- | --- |
| *Cladosporium* | Ascomycota; Dothideomycetes; Capnodiales | B01 | HQ696047 | *Cladosporium cladosporioidesstrain* Hu01 | 99 | Chen et al., 2012 EF405864 |
| *Cladosporium* | Ascomycota; Dothideomycetes; Capnodiales | B05 | HQ696045 | *Cladosporium* sp. MUT 4306 | 99 | Panno et al., 2013 KC339216 |
| *Cladosporium* | Ascomycota; Dothideomycetes; Capnodiales | B06 | HQ696044 | *Cladosporium* sp. TMS-2011 | 99 | Shrestha et al., 2011 HQ631003 |
| *Cladosporium* | Ascomycota; Dothideomycetes; Capnodiales | B08 | HQ696043 | *Cladosporium* sp. 7306 | 99 | Yarden et al., 2007 EF120415 |
| *Cladosporium* | Ascomycota; Dothideomycetes; Capnodiales | B09 | HQ696042 | *Cladosporium sphaerospermum* isolate wb311 | 99 | Buzina et al., 2003 AF455481 |
| *Cladosporium* | Ascomycota; Dothideomycetes; Capnodiales | B10 | HQ696041 | *Cladosporium* sp. 7306 | 99 | Shrestha et al., 2011 HQ631003 |
| *Cladosporium* | Ascomycota; Dothideomycetes; Capnodiales | B11 | HQ696040 | cf. *Cladosporium* sp. ATCAJ-13 | 99 | Lucero et al., 2011 HM596871 |
| *Cladosporium* | Ascomycota; Dothideomycetes; Capnodiales | B25 | HQ696029 | *Cladosporium cladosporioides* strain Hu01 | 99 | Chen et al., 2012 EF405864 |
| *Cladosporium* | Ascomycota; Dothideomycetes; Capnodiales | zzz409 | HQ696079 | *Cladosporium cladosporioides* strain LPSC1088 | 99 | Llorente et al., 2012 JF949719 |
| *Cladosporium* | Ascomycota; Dothideomycetes; Capnodiales | zzz1737 | HQ696055 | *Cladosporium* sp. TMS-2011 voucher SC8d50p14-1 | 99 | Shrestha et al., 2011 HQ631003 |
| *Aureobasidium* | Ascomycota; Dothideomycetes; Dothideales | B23 | HQ696031 | *Aureobasidium pullulans* | 98 | Ribeiro et al., 2009 FN428912 |
| *Alternaria* | Ascomycota; Dothideomycetes; Pleosporales | zzz407 | HQ696080 | *Alternaria tenuissima* isolate cwz-2 | 99 | Gao et al., 2011 HQ402558 |
| *Alternaria* | Ascomycota; Dothideomycetes; Pleosporales | zzz1740 | HQ696052 | *Alternaria longissima* strain ATCC 18552 | 95 | Pryor et al., 2000 AF229489 |
| *Curvularia* | Ascomycota; Dothideomycetes; Pleosporales | B34 | HQ696021 | *Curvularia* sp. M5 | 99 | Akita et al., 2011 HM371207 |
| *Leptosphaerulina* | Ascomycota; Dothideomycetes; Pleosporales | zzz511 | HQ696077 | *Leptosphaerulina chartarum* isolate ATT044 | 99 | Rodrigues et al., 2011 HQ607880 |
| *Phoma* | Ascomycota; Dothideomycetes; Pleosporales | B29 | HQ696025 | *Phoma* sp. 3 TMS-2011 | 99 | Shrestha et al., 2011 HQ631000 |
| *Phoma* | Ascomycota; Dothideomycetes; Pleosporales | zzz202 | HQ696085 | *Phoma* sp. TMS-2011 | 99 | Shrestha et al., 2011 HQ630963 |
| *Shiraia* | Ascomycota; Dothideomycetes; Pleosporales | B02 | HQ696046 | *Shiraia* sp. Z3 | 99 | Wu et al., 2013 JN198483 |
| *Shiraia* | Ascomycota; Dothideomycetes; Pleosporales | B17 | HQ696036 | *Shiraia* sp. JP185 | 95 | Morakotkarn et al., 2008 AB354994 |
| *Shiraia* | Ascomycota; Dothideomycetes; Pleosporales | B18 | HQ696035 | *Shiraia* sp. JP256 | 98 | Morakotkarn et al., 2008 AB354995 |
| *Shiraia* | Ascomycota; Dothideomycetes; Pleosporales | B22 | HQ696032 | *Shiraia* sp. slf14 | 99 | Zhu et al., 2010 GQ355934 |
| *Shiraia* | Ascomycota; Dothideomycetes; Pleosporales | B27 | HQ696027 | *Shiraia* sp. Z3 | 98 | Wu et al., 2013 JN198483 |
| *Shiraia* | Ascomycota; Dothideomycetes; Pleosporales | B33 | HQ696022 | *Shiraia* sp. Z3 | 99 | Wu et al., 2013 JN198483 |
| *Shiraia* | Ascomycota; Dothideomycetes; Pleosporales | zzz510 | HQ696078 | *Shiraia* sp. Z3 | 99 | Wu et al., 2013 JN198483 |
| *Shiraia* | Ascomycota; Dothideomycetes; Pleosporales | zzz613 | HQ696075 | *Shiraia* sp. Z3 | 98 | Wu et al., 2013 JN198483 |
| *Shiraia* | Ascomycota; Dothideomycetes; Pleosporales | zzz815 | HQ696073 | *Shiraia* sp. Z3 | 99 | Wu et al., 2013 JN198483 |
| *Shiraia* | Ascomycota; Dothideomycetes; Pleosporales | zzz816 | HQ696072 | *Shiraia* sp. Z3 | 99 | Wu et al., 2013 JN198483 |
| *Shiraia* | Ascomycota; Dothideomycetes; Pleosporales | zzz1021 | HQ696068 | *Shiraia* sp. Z3 | 99 | Wu et al., 2013 JN198483 |
| *Shiraia* | Ascomycota; Dothideomycetes; Pleosporales | zzz1023 | HQ696066 | *Shiraia* sp. Z3 | 99 | Wu et al., 2013 JN198483 |
| *Shiraia* | Ascomycota; Dothideomycetes; Pleosporales | zzz1225 | HQ696064 | *Shiraia* sp. LF15 | 99 | Wang et al., 2011 GU951760 |
| *Shiraia* | Ascomycota; Dothideomycetes; Pleosporales | zzz1226 | HQ696063 | *Shiraia* sp. Z3 | 99 | Wu et al., 2013 JN198483 |
| Undefined genus | Ascomycetes; Dothideomycetes; Pleosporales | B35 | HQ696020 | Pleosporales sp. JP225 | 99 | Morakotkarn et al., 2011 AB255301 |
| Undefined genus | Ascomycetes; Dothideomycetes; Pleosporales | zzz1429 | HQ696060 | Pleosporales sp. JP238 | 98 | Morakotkarn et al., 2006, AB255305 |
| Undefined genus | Ascomycetes; Dothideomycetes; Pleosporales | zzz1632 | HQ696058 | Pleosporales sp. JP7 | 99 | Morakotkarn et al., 2006 AB255241 |
| Undefined genus | Ascomycota; Dothideomycetes; | zzz714 | HQ696074 | Dothideomycete sp. 7667 | 99 | Feldman et al., 2008 EU680536 |
| *Penicillium* | Ascomycota; Eurotiomycetes; Eurotiales | B19 | HQ696034 | *Penicillium* sp. KH00315 | 99 | Sakayaroj et al., 2010 KH00315 |
| *Penicillium* | Ascomycota; Eurotiomycetes; Eurotiales | B32 | HQ696023 | *Penicillium sclerotiorum* isolate M9 | 99 | Yuan et al., 2006 HM595498 |
| *Penicillium* | Ascomycota; Eurotiomycetes; Eurotiales | B38 | HQ696018 | *Penicillium raistrickii* | 99 | Diguta et al., 2011 FR670335 |
| *Fusarium* | Ascomycota; Sordariomycetes; Hypocreales | zzz101 | HQ654261 | *Fusarium pulverosum* strain IBT 8051 | 99 | Yli-Mattila et al., 2004 AF414969 |
| *Fusarium* | Ascomycota; Sordariomycetes; Hypocreales | zzz305a | HQ696081 | *Fusarium proliferatum* NRRL 31071 | 99 | Kwon et al., 2001 AF291061 |
| *Fusarium* | Ascomycota; Sordariomycetes; Hypocreales | zzz612 | HQ696076 | *Fusarium polyphialidicum* isolate ATT183 | 96 | Rodrigues et al., 2011 HQ696076 |
| *Fusarium* | Ascomycota; Sordariomycetes; Hypocreales | zzz818 | HQ696071 | *Fusarium polyphialidicum* isolate ATT183 | 97 | Rodrigues et al., 2011 HQ607880 |
| *Fusarium* | Ascomycota; Sordariomycetes; Hypocreales | zzz1124 | HQ696065 | *Fusarium proliferatum* | 99 | Wicklow et al., 2009 GQ167232 |
| *Fusarium* | Ascomycota; Sordariomycetes; Hypocreales | zzz1327 | HQ696062 | *Fusarium proliferatum* NRRL 31071 | 99 | Kwon et al., 2001 AF291061 |
| *Fusarium* | Ascomycota; Sordariomycetes; Hypocreales | zzz1739 | HQ696053 | *Fusarium* sp. NRRL 25112 | 99 | O'Donnell et al., 2012 JF740892 |
| *Simplicillium* | Ascomycota; Sordariomycetes; Hypocreales | B26 | HQ696028 | *Simplicillium cylindrosporum* | 97 | Nonaka et al., 2013 AB603997 |
| *Colletotrichum* | Ascomycota; Sordariomycetes; Phyllachorales | B12 | HQ696039 | *Colletotrichum dematium* | 97 | Nirenberg et al., 2002 AJ301954 |
| *Colletotrichum* | Ascomycota; Sordariomycetes; Phyllachorales | B21 | HQ696033 | *Colletotrichum* sp. JP9 | 98 | Morakotkarn et al., 2010 AB255243 |
| *Colletotrichum* | Ascomycota; Sordariomycetes; Phyllachorales | B24 | HQ696030 | *Colletotrichum* sp. JP9 | 99 | Morakotkarn et al., 2006 AB255243 |
| *Colletotrichum* | Ascomycota; Sordariomycetes; Phyllachorales | B31 | HQ696024 | *Colletotrichum* sp. JP9 | 99 | Morakotkarn et al., 2006 AB255243 |
| *Colletotrichum* | Ascomycota; Sordariomycetes; Phyllachorales | B37 | HQ696019 | *Colletotrichum dematium* | 97 | Nirenberg et al., 2002 AJ301954 |
| *Colletotrichum* | Ascomycota; Sordariomycetes; Phyllachorales | zzz303 | HQ696084 | *Colletotrichum* sp. JP9 | 99 | Morakotkarn et al., 2006 AB255243 |
| *Colletotrichum* | Ascomycota; Sordariomycetes; Phyllachorales | zzz305 | HQ696082 | *Colletotrichum* sp. JP48 | 99 | Morakotkarn et al., 2006 AB255264 |
| *Colletotrichum* | Ascomycota; Sordariomycetes; Phyllachorales | zzz920 | HQ696069 | *Colletotrichum* sp. JP9 | 99 | Morakotkarn et al., 2006 AB255243 |
| *Colletotrichum* | Ascomycota; Sordariomycetes; Phyllachorales | zzz1428 | HQ696061 | *Colletotrichum dematium* | 97 | Nirenberg et al., 2002 AJ301954 |
| *Colletotrichum* | Ascomycota; Sordariomycetes; Phyllachorales | zzz1633 | HQ696057 | *Colletotrichum* sp. JP9 | 99 | Morakotkarn et al., 2009 AB255243 |
| *Colletotrichum* | Ascomycota; Sordariomycetes; Phyllachorales | zzz1738 | HQ696054 | *Colletotrichum* sp. JP9 | 98 | Morakotkarn et al., 2006 AB255243 |
| *Colletotrichum* | Ascomycota; Sordariomycetes; Phyllachorales | zzz1943 | HQ696049 | *Colletotrichum dematium* strain AR3563 | 97 | Farr et al., 2006 DQ286154 |
| *Arthrinium* | Ascomycota; Sordariomycetes; Xylariales | B16 | HQ696037 | *Arthrinium sacchari* strain FBC.045 | 100 | Mavragani et al., 2007 EF076711 |
| *Arthrinium* | Ascomycota; Sordariomycetes; Xylariales | B28 | HQ696026 | *Arthrinium* sp. 3 TMS-2011 | 98 | Shrestha et al., 2011 HQ630961 |
| *Arthrinium* | Ascomycota; Sordariomycetes; Xylariales | zzz304 | HQ696083 | *Arthrinium sacchari* strain FBC.045 | 99 | Mavragani et al., 2007 EF076711 |
| *Arthrinium* | Ascomycota; Sordariomycetes; Xylariales | zzz1022 | HQ696067 | *Arthrinium sacchari* isolate A09 | 98 | Gorfer et al., 2011 HQ115646 |
| *Arthrinium* | Ascomycota; Sordariomycetes; Xylariales | zzz1530 | HQ696059 | *Arthrinium arundinis* strain G41 | 99 | Bukovska et al., 2010 GU566268 |
| *Arthrinium* | Ascomycota; Sordariomycetes; Xylariales | zzz1842 | HQ696050 | *Arthrinium sacchari* isolate A09 | 99 | Gorfer et al., 2011 HQ115646 |
| *Monographella* | Ascomycota; Sordariomycetes; Xylariales | B13 | HQ696038 | *Monographella lycopodina* isolate LL | 99 | Jaklitsch et al., 2012 JF440979 |
| *Pestalotiopsis* | Ascomycota; Sordariomycetes; Xylariales | zzz2045 | HQ696048 | *Pestalotiopsis neglecta* isolate CCTU 12 | 100 | Arzanlou et al., 2013 JX854541 |
| *Xylaria* | Ascomycota; Sordariomycetes; Xylariales | zzz1741 | HQ696051 | *Xylaria arbuscula* isolate 89041211 | 99 | Hsieh et al., 2010 GU300090 |
| *Sebacina* | Basidiomycota; Agaricomycetes; Sebacinales | zzz919 | HQ696070 | *Sebacina endomycorrhiza* | 82 | Selosse et al., 2002 AF440650 |
| Undefined genus | Basidiomycota; Basidiomycetes | zzz1735 | HQ696056 | Basidiomycota sp. | 99 | Zhang et al., 1997 BSU65617 |

^a^ITS nrDNA sequences of cultural endophytic fungi were deposited at GenBank; ^b^Matches of ITS nrDNA sequences published in journals were also from GenBank.
